# Development and use of biomaterials as wound healing therapies

**DOI:** 10.1186/s41038-018-0139-7

**Published:** 2019-01-25

**Authors:** Rachael Zoe Murray, Zoe Elizabeth West, Allison June Cowin, Brooke Louise Farrugia

**Affiliations:** 1 The Institute for Health and Biomedical Innovation, School of Biomedical Sciences, Faculty of Health, Queensland University of Technology, Brisbane, QLD 4059 Australia; 2 Future Industries Institute, University of South Australia, Adelaide, SA 5095 Australia; 3 Graduate School of Biomedical Engineering, University of New South Wales, Sydney, NSW 2052 Australia

**Keywords:** Wound healing, Wounds, Burns, Biomaterials, Skin substitutes

## Abstract

There is a vast number of treatments on the market for the management of wounds and burns, representing a multi-billion dollar industry worldwide. These include conventional wound dressings, dressings that incorporate growth factors to stimulate and facilitate the wound healing process, and skin substitutes that incorporate patient-derived cells. This article will review the more established, and the recent advances in the use of biomaterials for wound healing therapies, and their future direction.

## Background

**Fig. 1:**
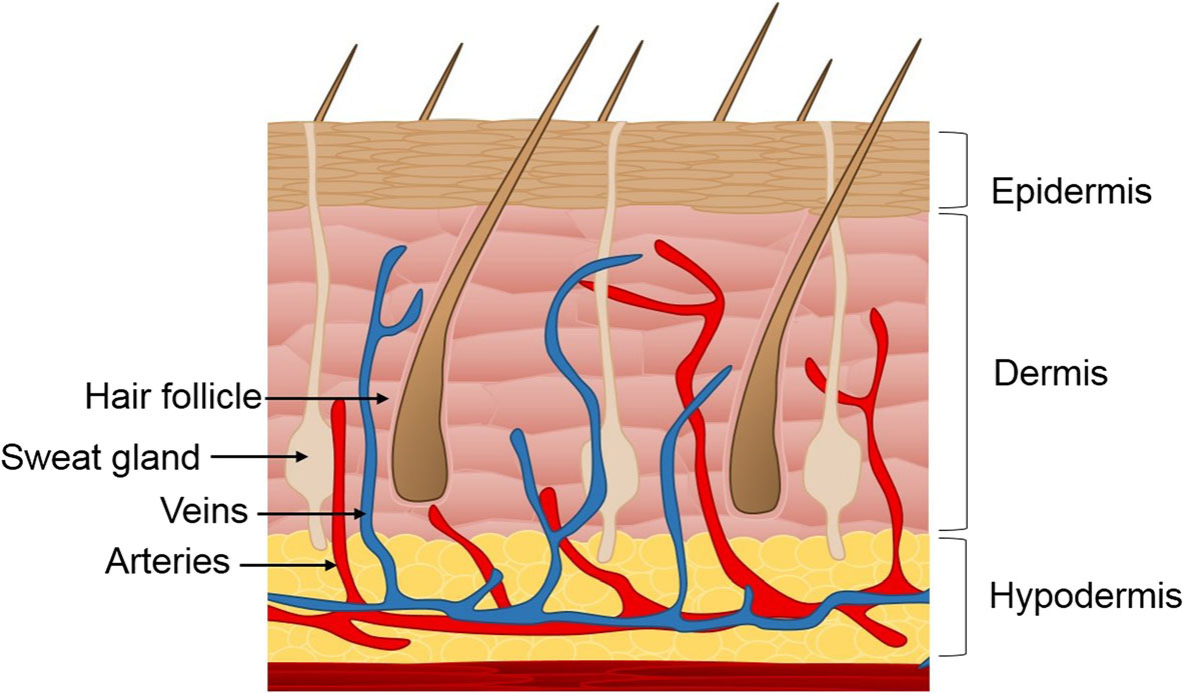
The three main layers of the skin: epidermis, dermis, and hypodermis

Skin plays a key role in protecting our internal environment from the external environment, maintaining homeostasis, and regulating temperature. On the outer side is the epidermis that consists predominantly of keratinocytes, which form a tight seal for protection (Fig. 1), along with melanocytes, Langerhan and Merkel cells [1]. Below this is the dermis, which is attached to the epidermis by the basement membrane, a thin layer of extracellular matrix (ECM) consisting mostly of laminins, integrins, perlecan, nidogen, and collagen IV [2, 3]. The composition of the dermis is complex and differs quite dramatically from the epidermis [1]. It consists of ECM, which acts as a scaffold for fibroblasts and other mesenchymal cells, blood vessels, hair follicles, and sweat glands [3–5]. It also houses molecules, such as growth factors and enzymes, that regulate the local environment [2, 3]. The dermis has several sublayers, with the papillary layer closest to the basement membrane consisting of poorly ordered thin collagen fibers housing a high density of fibroblasts [1]. Sandwiched between the lower dermal white adipose tissue and the papillary layer is the reticular dermis in which collagen fibers are thicker, more ordered, and sparsely populated with cells [1]. This complex nature of the skin makes it particularly difficult to replicate in the laboratory.

For many wounds, the healing process follows an ordered series of events including homeostasis, inflammation, proliferation/matrix deposition, and remodeling (reviewed in detail [1, 6]). For repair to occur, fibroblasts and other cells must fill the void created by the injury, with new blood vessels and ECM to form the granulation tissue, over which keratinocytes migrate to reseal the skin [6]. However, in cases such as burns where the damage to the epidermis and dermis can be extensive, the repair process is more complex. Here, cells and matrix to support the restoration of the skin are often reduced, or lacking, depending on the depth and severity of the injury. This leads not only to a slow healing process but also the potential for increased scar formation.

There is a vast number of treatments on the market for the management of wounds and burns [7], with the majority being wound dressings. Current wound dressings are comprised of a wide range of material types and claims with regard to what they treat. However, questions remain as to how well they facilitate the healing process [8]. Wound dressings, including films and foam dressings, are made from various materials, with some containing biologics or materials know to have antibacterial properties or agents that can facilitate cell migration. Additionally, there is a number of therapies currently on the market, such as skin substitutes derived from either de-epidermized tissue that can contain skin-derived cells, or alternatively cells, including fibroblasts and keratocytes, within a biological matrix or delivery vehicle [7], which will be described in more detail throughout the review.

## Review

### Wound dressings

**Fig. 2:**
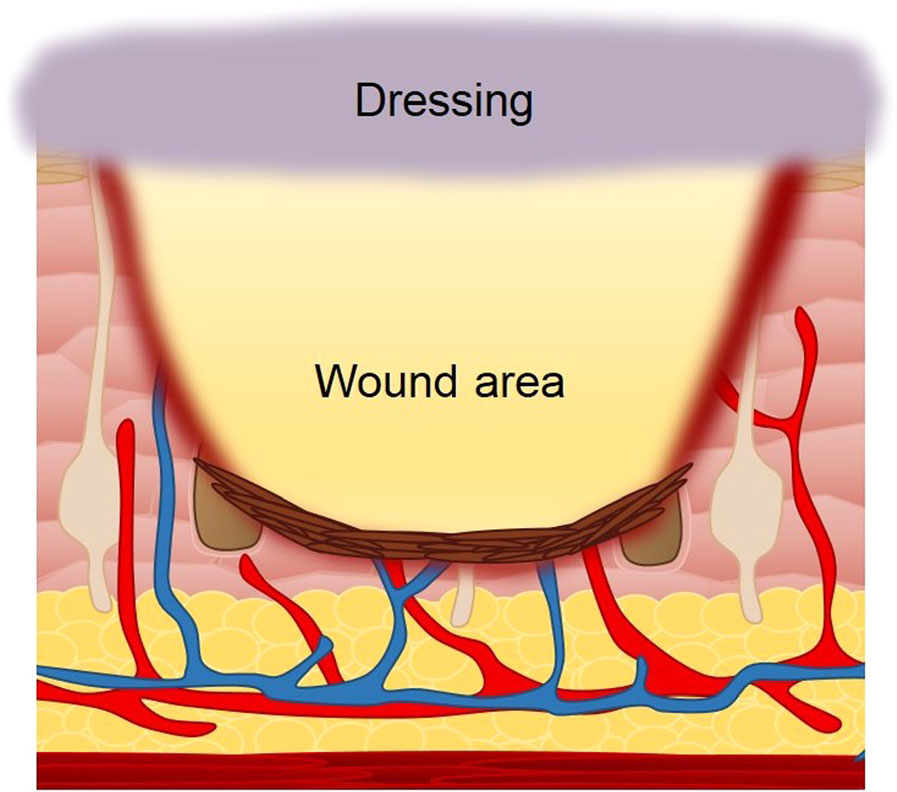
Schematic representation of a wound dressing, designed to create a sealed wound environment to keep out pathogens and promote the wound healing process

Wound dressings have been fabricated out of different types of materials and various formats, for example fiber mats and hydrogels, and may contain additivities like silver for anti-bacterial properties. Conventional wound dressings serve to create a sealed wound environment to keep out infection, while also creating a moist environment to promote the wound healing process (Fig. 2). Recent progresses in the development of advanced wound dressings has seen the use of materials and/or the incorporation of biologics capable of either stimulating or promoting events in wound healing, from cellular migration, to the production of ECM components [9].

#### Fiber mats

Conventional wound dressings were originally made from cotton gauze or non-woven blends of similar materials. Current research into wound dressings includes electrospun mats that create a coverage for the wound but enable the exchange of gases through the dressing. Fiber mats prepared from polymers, including polycaprolactone, often include incorporation of a biological material like collagen [10] to mimic the dermis. Incorporation of known antibacterial compounds including silver [11] and gentamicin [12] are an added feature of many of these dressings.

**Fig. 3:**
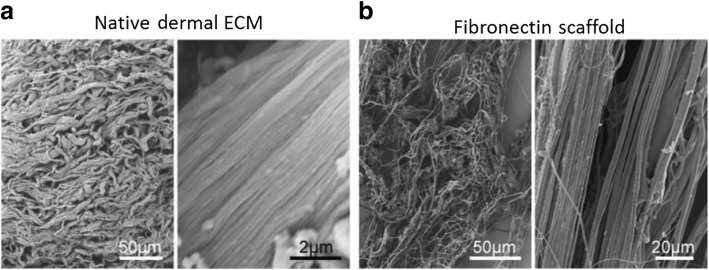
Scanning electron micrographs (SEMs) of the micro- and macro-structure of **a** native dermal extracellular matrix (ECM) and **b** fibronectin scaffolds for wound healing applications. Figure adapted with permission from the original article of Chantre et al. [13]. (Copyright 2018 by Elsevier Ltd)

One of the drawbacks of using synthetic materials, like polycaprolactone, as a wound dressing is that the dressing will eventually need to be removed, which may cause further damage to the wound. Fiber mats produced from natural materials, including dermal proteins, can be made to create wound dressings that mimic the ECM of the skin and can subsequently be incorporated into the body. Depending on the polymer/protein used, it may also stimulate wound healing responses. Fibronectin is one such protein found within the dermis and has been used to make scaffolds for potential wound healing therapies, which have been shown to not only accelerate wound healing but improve structural remodeling of the dermis and epidermis following healing [13]. The use of materials for the fabrication of scaffolds not only serves as material that biologically mimics the tissue that it is replacing, but it may also mimic the structure (Fig. 3).

#### Hydrogels

**Fig. 4:**
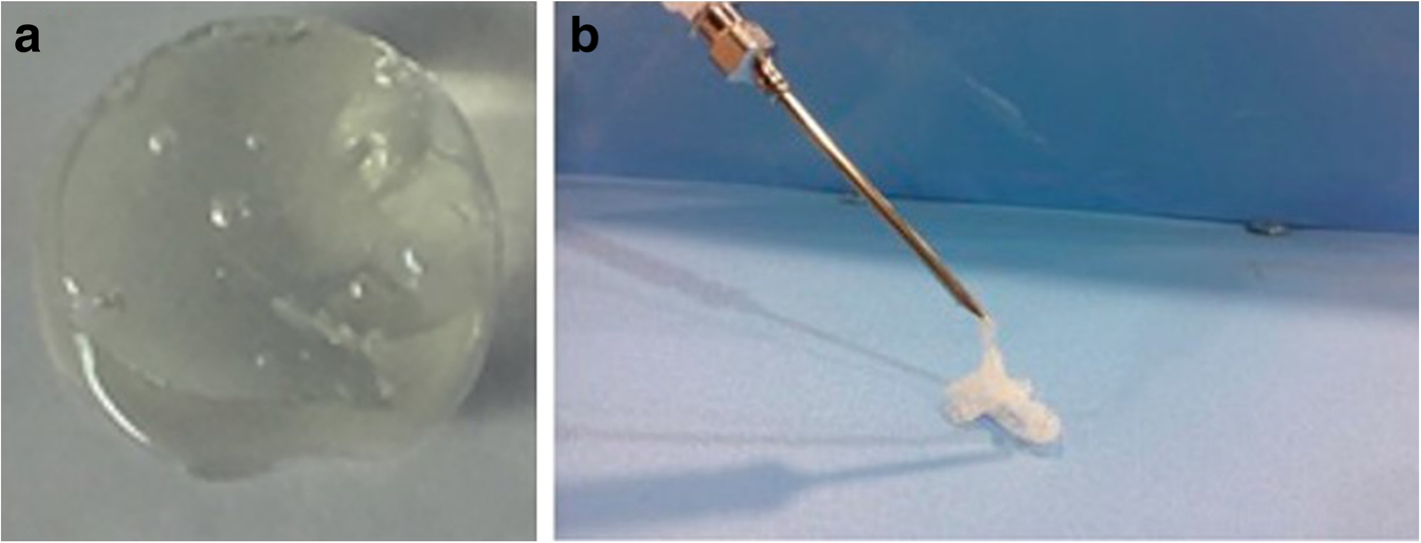
Various types of hydrogels have been, and are continuing to be used as wound healing therapeutics including hydrogels formed from the biopolymer **a** Hyaluronan hydrogel. Figure adapted with permission from the original article of Liyang et al. [25]. (Copyright 2017 by WILEY-VCH Verlag GmbH & Co. KGaA, Weinheim). **b** Injectable hydrogels show promise for wound healing applications. Figure adapted with permission from the original article of Liao et al. [20] (Copyright 2018 by American Chemical Society)

Hydrogels (Fig. 4a) are good candidates for wound dressings as they are able to form a barrier from pathogens, as well as create a hydrated environment to help promote the body’s own wound healing response [14]. Poly(vinyl alcohol) (PVA) is a polymer that is commonly used in the fabrication of hydrogels and is frequently used in wound healing applications. PVA is often used in medical applications as it is known for its anti-protein fouling properties and is relatively biologically inert [15]. PVA hydrogels for wound healing often include other materials to stimulate the wound healing response such as curcumin [16] or zinc oxide nanoparticles [17] for antibacterial properties, and phlorotannins, derived from brown algae, which have been shown to promote fibroblast migration [18]. A polymer similar to PVA, poly(ethylene glycol) (PEG), is also commonly used for the fabrication of hydrogels, where Polymyxin B conjugated to PEG [19] has been shown to be antibacterial, and when combined as a hybrid with alginate can promote wound regeneration [20]. Advances in hydrogel polymerization methods also enable the use of injectable hydrogels (Fig. 4b) [20], which can be directly delivered onto a patients wound enabling complete and customized coverage.

A well-known component present in skin is hyaluronan, also known as hyaluronic acid [21]. Hyaluronan is a polysaccharide and is commonly used in hydrogels for wound healing. Hydrogels composed of hyaluronic acid and chitosan have been used to deliver the angiogenic promoting growth factor vascular endothelial growth factor (VEGF) and have been shown to be both antibacterial and angiogenic, suggesting it might have potential as a wound healing therapeutic [22]. Furthermore, hydrogels that have incorporated hyaluronan have been shown to promote blood clotting [23] and possess antibacterial properties [24, 25]. Other polysaccharides, including chitosan, [26] alginate [27, 28], and cellulose [29], have also been used to fabricate hydrogels and have shown promise as wound healing therapeutics.

#### Wound dressings with incorporated biologics

In each of the different phases of wound healing, various growth factors and cytokines are involved in biological processes that result in the progression of the wound to the following healing phase. The harsh environment within a non-healing wound often results in either the absence of cells that produce and secrete the required growth factors and cytokines, or the degradation of those that are present. The delivery of growth factors and cytokines to wounds using biomaterials has been investigated not only for wound healing, but other regenerative applications. These require not only the incorporation of the growth factors and cytokines but also their delivery to the desired site of action in a functional and active state and at an appropriate concentration. *In vivo*, many growth factors are bound and protected by heparin/heparan sulfate [30] including members of the fibroblast growth factor (FGF) and VEGF families, and various cytokines that are associated with inflammation [31]. To mimic these *in vivo* interactions, heparin has been incorporated into wound healing therapeutics for the protection and delivery of growth factors, including VEGF [32] and transforming growth factor beta (TGFβ) [33]. Alternate methods for incorporation of growth factors include covalent incorporation [34], as well as genetically modified production of proteins to include incorporation of growth factors [35], or recombinant expression of growth factor fusion proteins [36] which can then be incorporated into biomaterial scaffolds for wound healing therapeutics. Additionally, the incorporation of exogenous growth factors or cytokines into biomaterial scaffolds has been shown to upregulate the expression of endogenous growth factors [37].

### Skin substitutes

There are three main types of skin substitutes available: dermal, epidermal, and dermal/epidermal [7]. Traditionally, skin substitutes, particularly dermal ones, have been composed of de-epidermized tissue, leaving the ECM as a scaffold, removing any components that could cause an immune response in recipients [7]. More recently, different types of skin constructs have been designed to mimic the ECM of the skin using components such as collagen, hyaluronan, and some have skin cells incorporated into them. Several commercially available skin substitutes, described in detail below, use xenogeneic components for example bovine collagen. While not ideal for use in products for human use, they are commonly used due to the lower cost, availability, and abundance as compared to human-derived components [38]. Technologies regarding recombinant protein production, particularly of human origin, is becoming more common with increasing presence in the research literature. This is likely to result in a reduction in associated costs with production and thus be translated into clinical use in the future [39].

#### Dermal substitute

Fibroblasts are found in every tissue of the body. In skin they are typically found embedded in the ECM, which forms the scaffold for the dermis [7]. Their role is to help maintain the structure and function of the dermis by continuously secreting growth factors, ECM precursors, and enzymes that modify these precursors. While they typically reside in the healthy dermis, they also migrate into wounds after injury [40, 41]. In the injured tissue, signals in the local environment cause fibroblasts to differentiate into myofibroblasts. One such signal is extra domain-A fibronectin which is not usually expressed under normal conditions but is upregulated after injury. In the wound, myofibroblasts play a key role in secreting ECM components, such as collagen and fibronectin, which form the scaffold necessary for cells to migrate into, and over, to populate the wound area [40, 41]. They also secrete growth factors, such as platelet-derived growth factor (PDGF) that modulate other cells in the wound, and enzymes, such as the matrix metalloproteinases and their inhibitors, that play key roles in remodeling the ECM and contribute to the final wound healing outcome. These same myofibroblasts are also responsible for the contractility of scar tissue as it matures [40, 41].

The role of myofibroblasts in the production and remodeling of the ECM, and in the contraction that drives fibrotic disease has led to extensive research into the nature and source of these cells. In skin, there are at least three populations of dermal fibroblasts that can exhibit different phenotypes depending on the location and age of the skin [4, 5, 42]. The papillary (superficial) dermal fibroblasts are found in the ridge-like structure of the papillary dermis. Below this are the reticular dermal fibroblasts and lastly there is a population that accumulates around hair follicles [5]. It should also be noted that dermal fibroblasts are not the only sources of myofibroblasts in the wound, for example mesenchymal stem cells found in the dermal sheath surrounding the hair follicle can also differentiate into wound myofibroblasts [4, 5, 43].

Given their role in secreting ECM products that build the scaffold for cells to repopulate the wound, it is not surprising that several skin substitutes contain fibroblasts, either from the patients themselves (autologous) or allogenic (neonatal) fibroblasts. How well these recapitulate the different types of fibroblasts found in the skin is unknown and as further research into the area develops, the efficacy of these skin substitutes will improve.

#### Autologous dermal skin substitutes

**Fig. 5:**
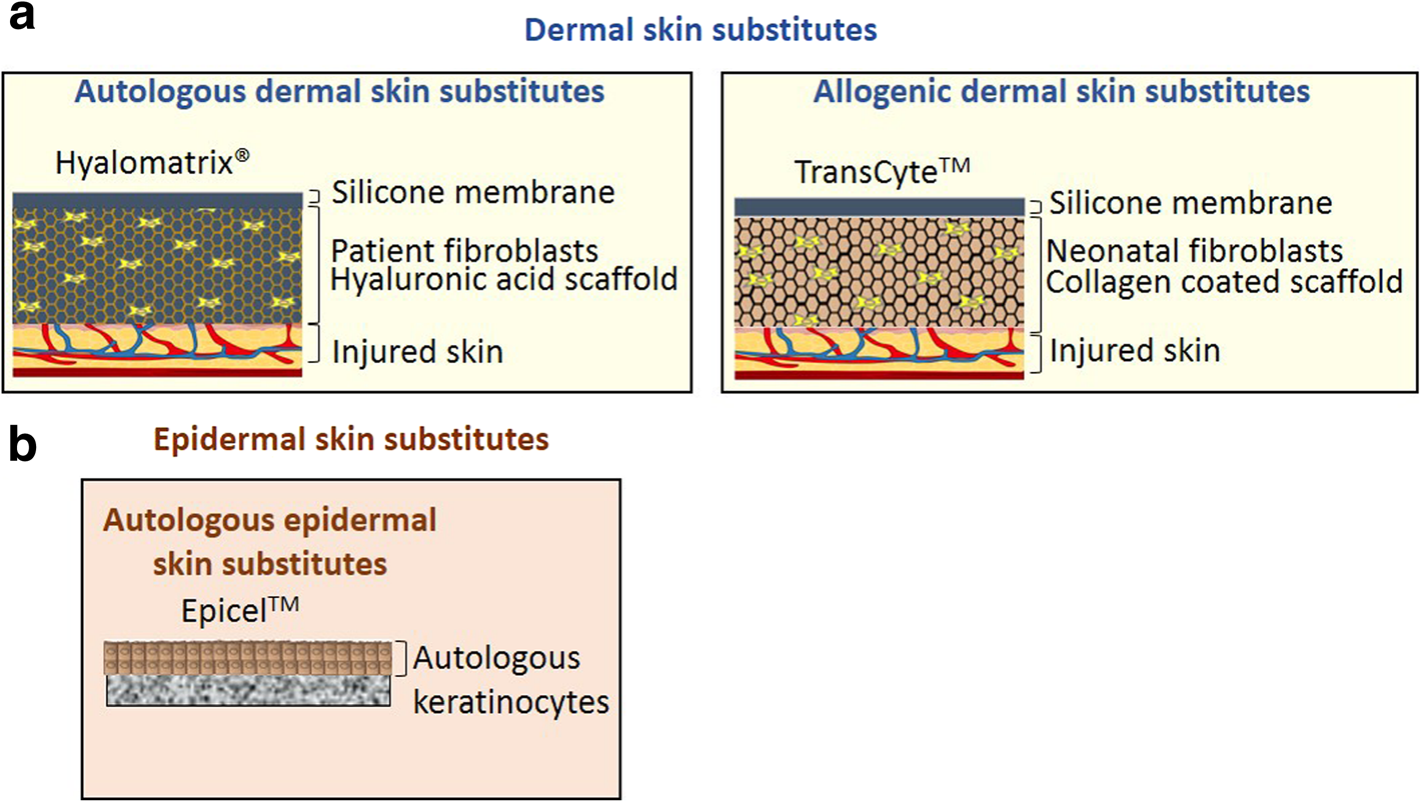
Schematic representation of **a** dermal skin substitutes and **b** epidermal skin substitutes

Hyaluronic acid is an anionic, non-sulphated glycosaminoglycan located in the ECM that promotes cell proliferation and migration of fibroblasts and keratinocytes [44]. The basal layer of the epidermis, where proliferating keratinocytes are located, has high levels of hyaluronic acid. Both Hyalograft three dimensional (3D) and Hyalomatrix® are hyaluronic acid-derived matrices that incorporate autologous fibroblasts [7, 45]. Hyalomatrix® (Fig. 5a), but not Hyalograft 3D, also has an outer silicone membrane that acts as a temporary epidermal barrier to protect the healing skin [7, 45]. The autologous fibroblasts secrete new ECM into the wound that “condition” the wound for split skin grafting. The main advantage of this skin substitute is that the cells are derived from the patient, which should minimize the immune response when applied to a wound. However, there needs to be a suitable donor site to collect the cells from the patient, and in vitro culture of these cells can take time before sufficient numbers are available for use, therefore, prolonging healing time for the patient.

#### Allogenic dermal substitutes

A number of dressings and skin substitutes, such as TransCyte™ and Dermagraft™, have been developed using scaffolds containing foreskin-derived neonatal fibroblasts [7, 45]. The concept behind these dressings is that the neonatal fibroblasts, although they are allogenic, are less immunogenic than adult fibroblasts. Importantly, like the autologous fibroblasts, they secrete new ECM and growth factors to aid the repair process [7]. TransCyte™ (Fig. 5a), a collagen-coated nylon matrix with an outer silicon film (no pores) seeded with human neonatal fibroblasts, has been used for both partial and full-thickness burn wounds [45]. Dermagraft™, used both for burns and chronic wounds, consists of a bioresorbable polyglactin scaffold containing human neonatal fibroblasts.

The key advantage of these types of dermal substitutes are that they are allogenic and can be applied immediately [45]. They are cryopreserved to maintain fibroblast viability, and so, unlike the autologous substitutes, there is no waiting period needed to grow enough patient cells to cover a wound. Dermagraft™ also has the advantage that there is no need for it to be removed from the wound, and thus, the typical “ripping off” of layers of newly forming skin does not occur as is seen with some dressing, particularly those fabricated from synthetic materials that are required to be removed. Cells and the scaffold material are not incorporated into the new skin that closes the wound, with the neonatal cells being non-viable long-term and within 3–4 weeks the polyglycolic acid mesh is absorbed and is no longer present in the wound. A common disadvantage of skin substitutes is the cost to patients that is associated with their production. As an example a single Dermagraft™ dressing is in the thousands of dollars [46], however, if successful only a single graft is required.

#### Epidermal substitutes

As highly specialized epithelial cells, the epidermal keratinocytes provide skin with the ability to act as a barrier to the external environment and help prevent dehydration. Roughly 90% of the epidermis consists of keratinocytes, with the basal keratinocytes housing many of the keratinocyte stem cells that continuously replenish the skin with its new layers [2, 47, 48]. The basal stem cells divide and many of these cells differentiate, eventually losing their organelles as they are continually pushed up, by the newer dividing cells, so they form the outer most layer, the stratum corneum. Since the first successful keratinocyte culture in the 1970s, these cells have been used to treat burns, either as allografts or autografts. Traditionally, they were typically transferred to the burn site as sheets of cells, but these sheets are fragile, and therefore, substitutes, such as EpiCel™, that provide a more stable surface for their transfer have been developed. EpiCel™ (Fig. 5b) is formed by growing a sheet of autologous keratinocytes to two to eight cells thick on mouse 3T3 fibroblasts, which takes around 16 days, and then the sheet of keratinocytes is attached to a petroleum gauze. This is then layered onto the wound and the gauze is removed 7 days later. It is around 50 cm^2^ but can still suffer from fragility when relocating it to the wound.

Basal keratinocytes with their organelles intact are the main cell type responsible for the re-epithelialization process after injury and contain the stem cells responsible for regeneration [2, 48]. Recently, keratinocytes have been used in gene therapy to treat the skin disease epidermolysis bullosa, which like some burns can lead to wounds covering a large surface area [49]. Keratinocytes were genetically modified to contain the wild-type LAM3B (laminin 332) gene and grown as sheets of cells containing approximately 4% holoclones (the stem cells) [49]. These sheets of cells were shown to restore skin integrity over 80% of the body and correct the defect as defined by the presence of laminin 332 in skin with no blister formation observed 2 years later [49]. More importantly, they showed through polymerase chain reaction and clonal tracing that transient amplifying progenitors have a half-life of 3–4 months and the regenerated skin was sustained only by these long-lived stem cells (holoclones) [49]. This is good news for the use of cultured epithelial autografts as it confirms that, when grown correctly, cultured epithelial autographs can restore skin integrity and are incorporated into the skin for life. However, it should be noted that the patient’s dermis was intact, while for many burns patients the dermis is reduced or missing after injury, so presenting a further challenge that is driving research into developing more epidermal/dermal substitutes.

#### Epidermal/dermal substitutes

**Fig. 6:**
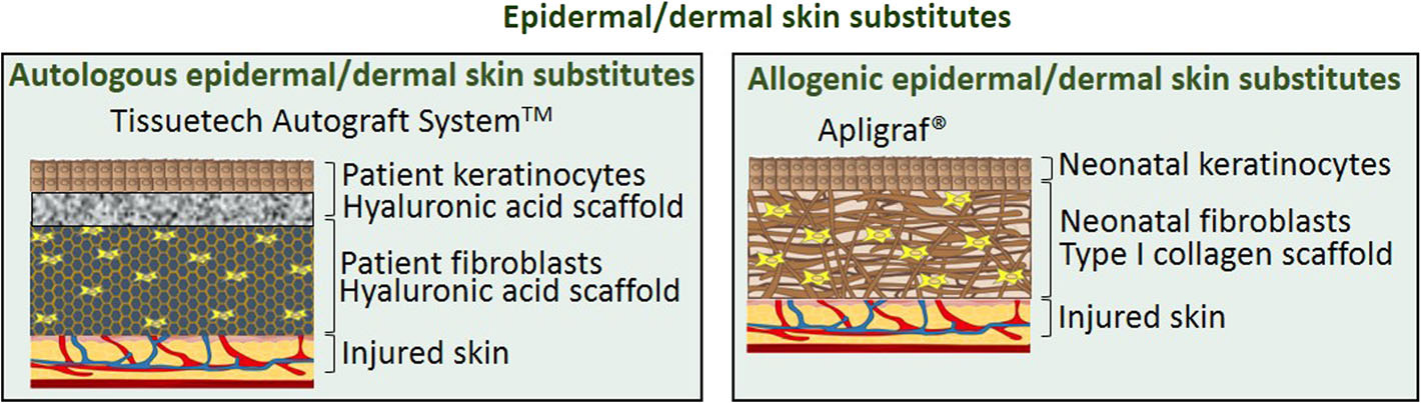
Schematic representation of epidermal/dermal skin substitutes

During the normal wound healing process, there is continuous cross talk between keratinocytes in the epidermis and fibroblasts (and other cells) in the dermis [6]. This communication, in the form of mediators such as growth factors, co-ordinates actions that restore tissue [6]. This, along with the lack of a dermis in some burns, has led to skin substitutes being designed around scaffolds that contain both keratinocytes and fibroblasts [7, 45] (Fig. 6). The idea being to more closely mimic the normal skin architecture and the communication that occurs between the dermis and the epidermis in the substitutes.

Apligraf® is one such example of an epidermal/dermal substitute [7, 45]. It is constructed using neonatal dermal fibroblasts grown in a matrix that consists of bovine-derived type I collagen with layers of human neonatal epidermal keratinocytes on top that have been exposed to air to promote stratification in order to mimic the stratum corneum. This upper layer then acts as an effective barrier to the environment. Another similar bilayer cellular substitute is OrCel™ where neonatal fibroblasts are cultured on one side of a bovine-derived type I collagen sponge and keratinocytes on the other side [7, 45]. The matrix is absorbed during the healing process, and according to the manufacturer, DNA from the allogenic cells is no longer present 2–3 weeks after application.

### Future directions

The heterogeneous nature of wounds, whether they are acute or chronic, the patients underlying pathologies, and the degree as to which the wound penetrates through the layers of the skin increase the complexity of developing a therapy that is appropriate for all wounds. Where the therapies detailed in this review are typically developed for a specific wound type, for example, Novosorb™, a biodegradable synthetic polymer, has been developed for burn patients with full-thickness wounds to a significant percentage of their body surface area (~ 20–50%) [50], whereas Apligraf™, produced from bovine collagen and human-derived cells, is for the treatment of chronic venous leg ulcers and diabetic foot ulcers, and while the existing dressings and skin substitutes are good, they can be improved. The ECM, in addition to providing a scaffold for cells to adhere to and migrate on, provides mechanical stability and biochemical cues that play roles in tissue homeostasis and during the repair process [51]. It is comprised of over 300 proteins, 200 glycoproteins, and 30 proteoglycans, and so its exact composition, which can differ over time and under different circumstances, such as inflammation and after injury, can alter the outcome of the repair process. The ECM, and the growth factors housed within it, interacts with cells, triggering signaling pathways that can lead to proliferation, cell motility, or stasis depending on its composition. Our understanding of the composition of the ECM, and how the presence of specific combinations of proteoglycans can alter its structure and function, is relatively limited compared to what is known about the composition and formation of the epidermis. While there is no doubt that neonatal fibroblasts produce ECM that is beneficial to the repair process, whether the neonatal fibroblasts produce an ECM composition that is the “best” for wound healing or whether it can be fine-tuned to make the cells produce additional ECM components and growth factors that will improve the process is yet to be fully elucidated. One of the challenges that needs to be tackled is the ability to recreate the complexity of the dermis. The development of biomaterials going forward for wound healing therapies will need to approach these issues of creating an environment that closely resembles that of native skin, where materials in the future should mimic those present in the dermis in terms of their structure as well as biologic functionality. Current and future research will help answer these questions and aid the development of both dressings and skin substitutes to improve burn wound healing.

Along with the development of materials and technologies to more economically produce materials for wound healing therapies, technologies for the fabrication of scaffolds that use these materials have too advanced in recent years. The ability to manufacture scaffolds using 3D printing technologies has enabled the development of skin substitutes that not only can be produced to be specific to patient wounds but also the use of bioinks that allow the printing of scaffolds laden with cells [52]. Furthermore, advances in bioprinting and bioinks now enable the direct printing of scaffolds onto parts of the body, opening up the ability to print scaffolds directly onto patients wounds in the future [53]. Additionally, the ability to print scaffolds that can be fabricated to contain multiple layers consisting of different materials and laden with different cell types is a step towards being able to approach the challenge of creating the heterogenous structure of skin in the laboratory.

For burns patients, the ability to collect skin for autografts can be limited by the area of the burn and the sites that containing healthy skin. This has led to research into other sources of stem cells [2]. Hair follicles are easily accessed and contain stem cells capable of differentiating into and restoring skin after grafting [47]. EpiDex™ is an autologous epidermal equivalent generated from follicular stem cells (out root sheet cells) taken from patient’s hair. Stem cells from 50 to 200 hairs plucked from patients are cultured on a microporous membrane with fibroblast feeder layer of growth-arrested human dermal fibroblasts on the lower side. The cells are then detached from the microporous membrane and attached to a silicone membrane ready for use. The disadvantage here is the size of the EpiDex™, which is 1 cm^2^, making it unsuitable for large burns. Further research is needed to develop larger grafting material, incorporation of stem cells from different populations, or using induced pluripotent stem cells derived from blood cells that are reprogrammed back into an embryonic-like pluripotent state that permits these cells to then differentiate into keratinocytes or fibroblasts.

When the dermis and epidermis are lost due to a burn injury, some of the structures typically found in these areas are more often not replaced during the repair process. This includes hair follicles and sweat glands. This means that the skin that regenerates is generally hairless and does not sweat properly. No epidermal/dermal substitute has been developed yet that contains structures such as hair follicles or sweat glands. Also missing from scar tissue are melanocytes, the cells that produce pigments that give the skin its color. No skin substitutes to date contain these cells, but research in mice using skin substitutes containing melanocytes suggest that skin tone can be regained [54]. Incorporation of adipose-derived stem cells into a recombinant collagen scaffold demonstrated superior wound healing when compared to the recombinant protein scaffold alone [55]. The ability to incorporate stem cells that are able to differentiate into various lineages, depending on their environment, coupled with material scaffolds that are able to facilitate these environment ques, show enormous promise in their ability to facilitate wound healing and direct the next generation of wound healing therapies [56].

## Conclusions

This review details a variety therapies that are currently available to patients for the treatment of wounds and burns that incorporate a biomaterial component. These therapies range from polymer hydrogels to epidermal/dermal substitutes that incorporate both keratinocytes and dermal fibroblasts. Due to the heterogeneous nature of wounds, there is no “one fits all” therapy, though the continual advancement in technologies used to develop these therapies, from 3D printing of dressings directly onto a wound, to stem cell technologies including induced pluripotent stem cells, will result in new wound healing therapies in the future.

## Abbreviations

ECM: Extracellular matrix
FGF: Fibroblast growth factor
PDGF: Platelet-derived growth factor
PEG: Poly(ethylene glycol)
PVA: Poly(vinyl alcohol)
TGFβ: Transforming growth factor beta
VEGF: Vascular endothelial growth factor
